# Vascular Dysfunction in a Mouse Model of Rett Syndrome and Effects of Curcumin Treatment

**DOI:** 10.1371/journal.pone.0064863

**Published:** 2013-05-21

**Authors:** Anna Panighini, Emiliano Duranti, Ferruccio Santini, Margherita Maffei, Tommaso Pizzorusso, Niccola Funel, Stefano Taddei, Nunzia Bernardini, Chiara Ippolito, Agostino Virdis, Mario Costa

**Affiliations:** 1 Institute of Neuroscience, Italian National Research Council (CNR), Pisa, Italy; 2 Scuola Normale Superiore, Pisa, Italy; 3 Department of Endocrinology and Kidney; University-Hospital of Pisa, Pisa, Italy; 4 Dulbecco Telethon Institute, Rome, Italy; 5 Institute of Food Science, CNR, Avellino, Italy; 6 Department of Clinical and Experimental Medicine, University of Pisa, Pisa, Italy; 7 Department of Surgery, University of Pisa, Pisa, Italy; 8 Institute of Psychology, University of Florence, Florence, Italy; University of Insubria, Italy

## Abstract

Mutations in the coding sequence of the X-linked gene MeCP2 (Methyl CpG–binding protein) are present in around 80% of patients with Rett Syndrome, a common cause of intellectual disability in female and to date without any effective pharmacological treatment. A relevant, and so far unexplored feature of RTT patients, is a marked reduction in peripheral circulation. To investigate the relationship between loss of MeCP2 and this clinical aspect, we used the MeCP2 null mouse model B6.129SF1-MeCP2tm1Jae for functional and pharmacological studies. Functional experiments were performed on isolated resistance mesenteric vessels, mounted on a pressurized myograph. Vessels from female MeCP2^+/−^ mice show a reduced endothelium-dependent relaxation, due to a reduced Nitric Oxide (NO) availability secondary to an increased Reactive Oxygen Species (ROS) generation. Such functional aspects are associated with an intravascular increase in superoxide anion production, and a decreased vascular eNOS expression. These alterations are reversed by curcumin administration (5% (w/w) dietary curcumin for 21 days), which restores endothelial NO availability, decreases intravascular ROS production and normalizes vascular eNOS gene expression. In conclusion our findings highlight alterations in the vascular/endothelial system in the absence of a correct function of MeCP2, and uncover related cellular/molecular mechanisms that are rescued by an anti-oxidant treatment.

MeCP2 (Methyl CpG-binding protein) is a X-linked epigenetic transcriptional regulator abundant in pericentric heterochromatin and widely distributed in a variety of cell types and tissues including circulatory system and endothelial cells [1], [2], [3], [4]. Mutations in the MECP2 gene are the major documented cause of Rett Syndrome (RTT), a neurological disorder with a strong social impact due to its relatively high prevalence in the population (1/10000 live female births), which makes this disease one of the most common causes of intellectual disability in females [1], [5], [6]. Affected children develop normally until 6–18 months of age, after which time they rapidly regress in purposeful hand use and spoken language, with the development of gait abnormalities and hand stereotypies [4]. Furthermore they present severe progressive anomalies, such as autistic features, seizures, developmental delay, loss of acquired motor skills and speech [7], [8], [9], [10]. In addition to the prominent neurological symptoms, children with RTT frequently present reductions in skeletal growth, hypo-perfusion in the area of midbrain and upper brainsteam, constipation, contracted joints, and poor circulation, which lead to bluish tints to their feet and legs [11], [12]. Hydrotherapy and physiotherapy to the extremities is often used to regain proper circulation and helps to keep their extremities limber [13].

Different mouse models of Rett Syndrome have been generated by early embryonic deletion of the gene encoding MeCP2 [14], [15], [16], [17]. All these mouse models present a clear RTT phenotype that recapitulates what observed in patients[18], [19] [20], [21], [22]. The MeCP2^tm1Jae^ male mice, herein utilized, exhibit a significantly reduced body weight, a feature dependent on genetic background and indicating the presence of genes acting on body weight and downstream of MeCP2 [14], [23]. The MeCP2^tm1Jae^ male mice present also body tremor and shaking paw by the fifth week, while heterozygous mutant females develop normally and are apparently asymptomatic until the age of four months, when they begin to show pathological symptoms including reduced activity, ataxic gait, piloerection, stereotyped movements and heavy breathing [22].

While the neurological phenotype of the Rett syndrome has been well-characterized in animal models and in humans, the alterations in the cardiovascular system of MeCP2 deficient mice have been poorly investigated [24], [25] leaving peripheral vascular functional aspects completely unexplored.

In the last 2 decades, a large body of evidences clearly documented the critical role played by endothelium in the modulation of vascular tone and structure, mainly by the production and release of nitric oxide (NO), which derives by the activity of the constitutive endothelial enzyme NO synthase (eNOS) [26], [27]. Interestingly, previous work obtained *in vitro* showed a different recruitment of MeCP2 to the eNOS promoter in human endothelial and vascular smooth muscle cells [28], [29].

To date, no effective pharmacological treatment exists for RTT, neither for the central nervous system nor for peripheral symptoms. Curcumin (diferuloylmethane) is a natural polyphenol compound known to interact with multiple targets and used for centuries to treat a large number of diseases. Micro molar plasma concentration of curcumin results in anti-oxidant (through scavenging of free radicals), anti-inflammatory and cardiovascular properties; lower concentration of curcumin (500 nM) results in a neurological protective effects increasing the proliferation of neural stem cells in hippocampus [30], [31], [32]. It has been shown that curcumin protects the vascular endothelium, by the production of endothelial heme-oxigenase [33], [34], while in Alzheimer disease it counteracts β-amyloid-induced oxidative stress[34] [35]. Some of these observations can be explained by the several activating and inhibitory actions of the drug on a number of key signaling pathways, including MAP Kinase, Protein kinase C and BDNF. Additionally, given its potent activity on histone deacetylases (more potent that sodium butyrate or valproic acid [36], [37]), curcumin may be considered an epigenetic drug, this being of potential relevance in the absence of a correct MeCP2-based chromatin remodelling, like that postulated for RTT syndrome.

The present study was designed to assess endothelial function in RTT mice. Specifically we investigated whether the resistance of mesenteric vessels of symptomatic heterozygous MeCP2 knockout female and hemizygous male (MeCP2^+/−^, MeCP2^y/−^) mice display an altered endothelium-dependent relaxation vascular ROS generation, NO availability, and vascular eNOS expression. The same parameters were analyzed also following curcumin treatment, to explore the capacity of an- anti-oxidant “epigenetic” drug to rescue the pathological phenotype. Our results demonstrate that mesenteric resistance arteries from RTT mice are characterized by endothelial dysfunction caused by a reduced NO availability secondary to an increased ROS generation, associated with an intravascular increase in superoxide anion production, and a decreased vascular eNOS expression. These alterations are reversed by curcumin administration, which restores endothelial NO availability, decreases intravascular ROS production and normalizes vascular eNOS gene expression.

## Materials and Methods

### Animals

All experiments were carried out in accordance with the directives of Council of European Communities (86/609/EEC) and approved by Italian Ministry of Health for the care and use of laboratories. The animals used for experiments were derived from heterozygous B6.129SF1-*MeCP2^tm1Jae^* knock-out females (MeCP2^+/−^) [15]. The animals are maintained through heterozygous females, this because the homozygous unfertile males present a dramatic phenotype and die within 2 months of age. Conversely, heterozygous females are fertile, present a certain degree of variability in the phenotype (due to the X-inactivation patterns) resembling in this regard more closely the human pathological condition [38]. Heterozygous females were originally crossed to C57BL6 for one generation, followed by breeding among offspring of the same generation with breeder changes and were maintained on a mixed background. Mixed background reduced mortality and was necessary to obtain the high numbers of mice required by extensive analysis. Age-matched female and male littermates were used in all experiments to control for possible effects of genetic background unrelated to the MeCP2 mutation [39], [40], [41]. Mutant and WT mice were reared in standard conditions. All experiments were performed on animals with clear symptoms that correspond to six weeks and six months of age for male (MeCP2^−/y)^ and female (MeCP2^+/−^). respectively [22]. The age discrepancy was inevitable since male mice are symptomatics since birth and die within 2 months of age, while heterozygous females start becoming symptomatic at 5 months of age, while before are not distinguishable from WT[14].

### Curcumin treatment

Six months old MeCP2^+/−^ mice and age-matched WT female littermates were distributed into homogeneous experimental groups (n = 9) taking into account age, weight (Table S1), and coordination capability (Rotarod test). MeCP2^+/−^ and WT received 5% (w/w) dietary curcumin or not for 21 days, as previously reported [42], [43]. Doses of curcumin were chosen according to previously tested clinical doses well tolerated in humans [44]. Age-matched MeCP2 ^+/−^ and WT female mice (n = 5 each) were treated with vehicle for 21 days, as a time-control study. Rotarod was used to assess the coordination capabilities of the animals. This was necessary to create symptomatically matched groups of mice for curcumin or vehicle treatment. We tested the time spent by the animal on the rotating rod under continuous acceleration from 4 to 40 rpm. The latency to fall off the rotating rod was used as an indication of motor coordination and balance. The task was repeated five times to evaluate the learning and memory capability.

Vehicle treated mice were not analysed simultaneously to curcumin treated groups and were added as an assessment of the natural history of the disease.

During the treatment animals were weighed once a week. After treatment, animals were sacrificed by cervical dislocation.

### HPLC evaluation of plasmatic curcumin

Blood from control and treated animals was isolated by cardiac puncture and curcumin standard solution was obtained according to [45]. Briefly, 150 µl of EDTA-stabilized mouse plasma was mixed with 50 µl of standard curcumin solutions (range 0.1 to 50 µg) of methanol dissolved curcumin (Sigma). Mouse plasma samples or curcumin standard solution were then measured by HPLC analysis in a Waters Alliance 2690 HPLC apparatus and data analysis was performed with Waters Millenium software.

*Liquid Chromatographic Conditions*: 50 µl sample or standard solution were injected on to a C18 column 3.9×150 mm, 4 µm particle size, Nova -Pak, Italy), fitted with a security Guard C18 cartridge column (I.D. 4.0 mm×3.0mm, Phenomenex srl, Bologna, Italy). The analysis time was 10 min per sample and the detection wavelength was 420 nm. The mobile phase was composed of acetonitrile 5% acetic acid (75∶25, v/v) at a flow rate of 1.0 mL/min. The mobile phase was filtered through a 0.45 µm nylon membrane filter and ultrasonically degassed prior to use.

### Preparation of Small Mesenteric Arteries for Reactivity Experiments

A second-order branch of the mesenteric arterial tree (≈2 mm in length) was dissected and mounted on 2-glass microcannule in a pressurized myograph, as described previously [46]. Vessels were equilibrated for 60 min under constant intraluminal pressure (45 mm Hg) in warmed (37°C) and bubbled (95% air and 5% CO_2_) physiological salt solution, which contained (in mmol/L): NaCl 120, NaHCO_3_ 25, KCl 4.7, KH_2_PO_4_ 1.18, MgSO_4_ 1.18, CaCl_2_ 2.5, EDTA 0.026, and glucose 5.5 (pH 7.4). Vessels were considered viable and used if they constricted ≈70% of their resting lumen diameter in response to an extraluminal application of high-potassium (125 mmol/L of KCl) physiological salt solution containing 100 µmol/L of noradrenaline (NA). In small arteries from all groups of animals endothelium-dependent relaxation was assessed by measuring the dilatory response to cumulative concentrations of acetylcholine (0.001–100 µM, Sigma Chemicals) in vessels pre-contracted with norepinephrine (10 µM). In preliminary experiments, we performed a concentration-response analysis of NA effects (from 1 nM to 100 µM) to establish at which level this compound was able to elicit similar contractions in vessels from control and mutant mice. After the titration study, the dose of 10 µM NA, which induced similar contracting responses in both groups, was selected (Table 1).

**Table 1:** Vascular parameters.

	Lumen diameter ( µm)	VC to NE (10 µM)
Female WT Baseline	219.8±30.4	54.8±1.9
Male WT Baseline	215.5±25.7	55.9±2.5
MeCP2^+/−^ Baseline	208.6±24.5	57.8±2.4
MeCP2^y/−^ Baseline	197.2±21.6	54.4±4.1
Female WT Curcumin	221.7±27.8	55.7±4.1
MeCP2^+/−^ Curcumin	207.4±26.8	56.7±3.8
Female WT Vehicle	226.2±35.6	54.1±5.3
MeCP2^+/−^ Vehicle	213.6±21.7	57.9±4.6

Table 1. vascular parameter of the mice employed in the curcumin experiment.

Vasoconstriction (VC) norepinephrine (NE).

To exclude the possibility that impaired relaxation in response to acetylcholine resulted from a muscarinic receptor defect, in additional groups of animals (n = 4 per group), a concentration-response curve to bradykinin (0.001–1 µM, Sigma Chemicals), an endothelial agonist acting through a different receptor and signal transduction pathway,[47] [48] was evaluated. Endothelium-independent relaxation was assessed by the dilatory response to sodium nitroprusside (0.01–100 µM, Sigma). To evaluate NO availability, a curve to acetylcholine was constructed after 30 min pre-incubation with the NOS inhibitor L-NAME (100 µM, Sigma). To evaluate the influence of ROS, acetylcholine was repeated in the presence of the antioxidant ascorbic acid (100 µM, Sigma, 30-min pre-incubation). Finally, to assess whether ROS generation can impair NO-mediated endothelium-dependent relaxation, acetylcholine was infused during simultaneous incubation with L-NAME and ascorbic acid.

### In situ Detection of Superoxide Anion

The *in situ* production of superoxide anion from 30 µm frozen mesenteric vessel sections was evaluated by means of the fluorescent dye dihydroethidium (DHE, Sigma), as previously described [49]. Three slides per segment were analyzed simultaneously after incubation with Krebs solution at 37°C for 30 min. Krebs-HEPES buffer containing 2 µM DHE was then applied to each section and evaluated under fluorescence microscopy.

In the presence of superoxide, DHE is oxidized and it intercalates in cell DNA, thus staining the nucleus with red fluorescence (excitation at 488 nm, emission 610 nm). A second acquisition was performed to detect the autofluorescence of elastin. In the whole artery section, the percentage of arterial wall area stained with the red signal was normalized to the total area examined and quantified by means of an image analysis software (McBiophotonics Image J; National Institutes of Health, Bethesda, MD).

### Determination of Plasma Malondialdehyde Assay

Serum was separated by centrifugation and stored at −80°C until assay. The colorimetric assessment of malondialdehyde level was performed according to the technical procedure of the kit (Cayman Chemical).

### RNA extraction and Real Time PCR

Total RNA was isolated from mesenteric vessels with Tripure (Roche Molecular Biochemicals). First-strand cDNA synthesis was performed using High Capacity cDNA Reverse Transcription Kit (Applied Biosystems). Taq-Man quantitative PCR (50°C for 2 min, 95°C for 10 min, followed by 40 cycles of 95°C for 15 s 60°C for 1 min) was used to eNOS mRNA expression. GAPDH was used as the invariant control. The primers used were all purchased from Applied Biosystems and reactions were run on a ABI PRISM® **7700** Sequence Detection System

### Immunostaining of eNOS

Small mesenteric arteries were immediately fixed in cold 4% paraformaldehyde after collection from small bowel loops and paraffin embedded at 56°C. Eight-micron-thick sections were serially mounted on glasses and sequentially treated as follows: 1% hydrogen peroxide in methanol for 30 minutes; microwave antigen retrieval (600 W in 10 mmol/L of sodium citrate; pH 6.0); rmal goat serum (1∶20; Dakopatts Glostrup, Denmark), rabbit anti-eNOS polyclonal antibody (sc-654, 1∶100 overnight at 4°C, Santa Cruz Biotechnology, Inc., Santa Cruz, CA), biotinylated anti-rabbit immunoglobulins, peroxidase-labeled streptavidin complex (Vector Laboratories, Burlingame California), 3,3-diaminobenzidine tetra-hydrochloride (DAB; Amresco, Solon, OH, USA), hematoxylin counterstaining. The specificity of eNOS immunostaining was assessed by preadsorbing the primary antibody with eNOS blocking peptide (sc-654 P, Santa Cruz Biotechnology), at 10 times the antibody concentration for 24 h at 4°C. Negative controls were obtained by substituting the primary antibody with preimmune rabbit serum. Endogenous peroxidases and avidin-binding activity were assayed by incubating slides with DAB alone or with peroxidase-labeled streptavidin complex/DAB, respectively, as previously reported [46]. The slides were examined under light microscope equipped with the digital camera DFC480 (Leica, Cambridge, United Kingdom). In the whole artery section, the percentage of labelled wall area was quantified by means of an image analysis software (McBiophotonics Image J) and normalized to the total area examined, as previously reported [50].

### Data Analysis

Results are presented as mean±SEM. The statistical significance of relaxation responses was assessed taking into consideration time course, treatment and gender by two-way ANOVA. Response curves comparison within each group were made by repeated-measures ANOVA or one-way ANOVA followed by a Student Newman-Keuls test, where appropriate. A value of *P*<0.05 was considered statistically significant. Maximal acetylcholine- and sodium nitroprusside-induced relaxant responses (E_max_) were calculated as maximal percentage increments of lumen diameter.

## Results

### Vascular Functional Experiments

**Figure 1:**
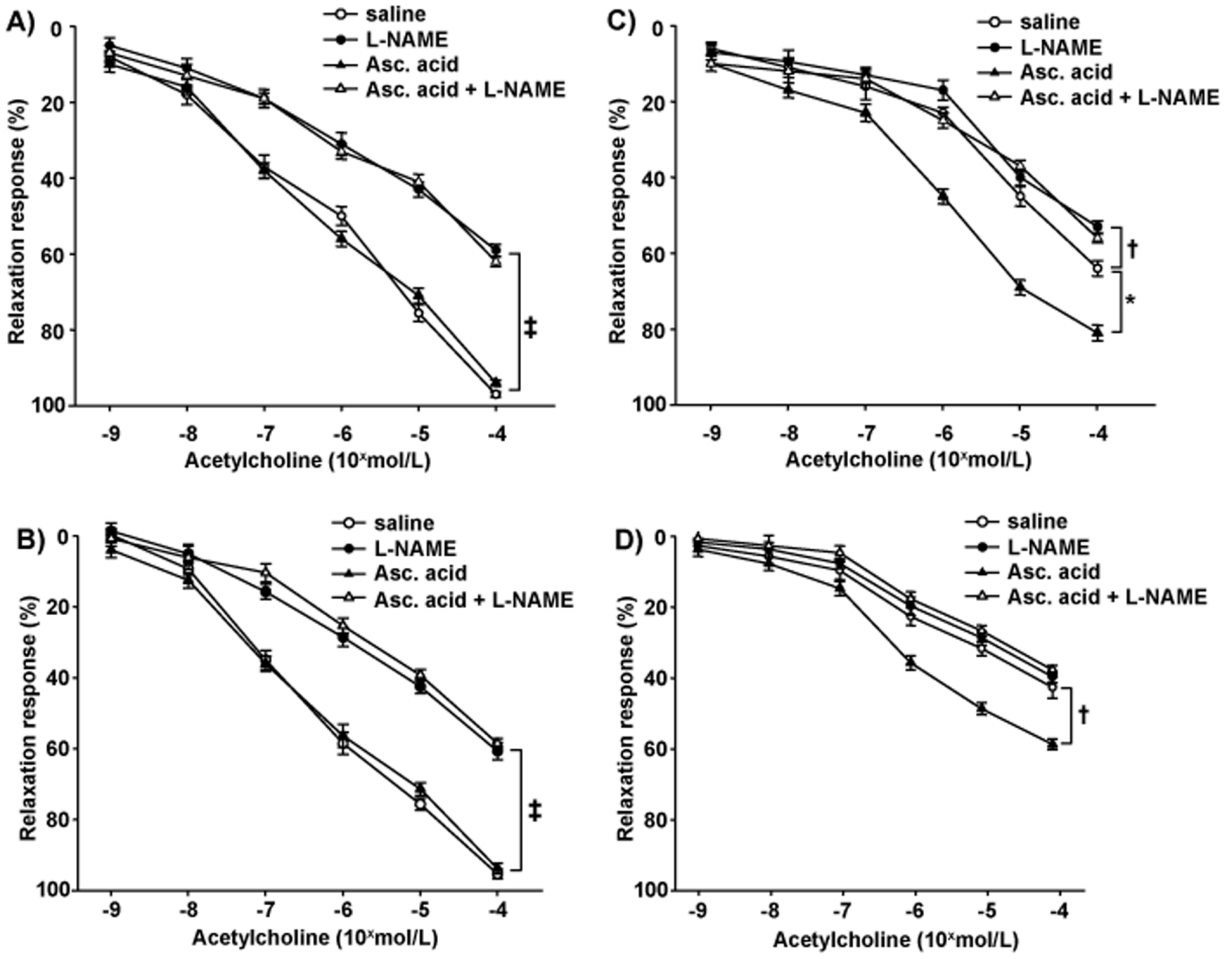
Endothelial relaxation in WT and KO mice. Relaxations to acetylcholine in mesenteric resistance arteries, without (saline) or with L-NAME, ascorbic acid, or both, in WT female mice (panel A) and WT male mice (panel B), MeCP2^+/−^ female mice (panel C), or MeCP2^−/y^ male mice (panel D). Results on 9 animals are given as mean±SEM. *P<0.01; †*P*<0.05; ‡*P*<0.001.

**Figure 2:**
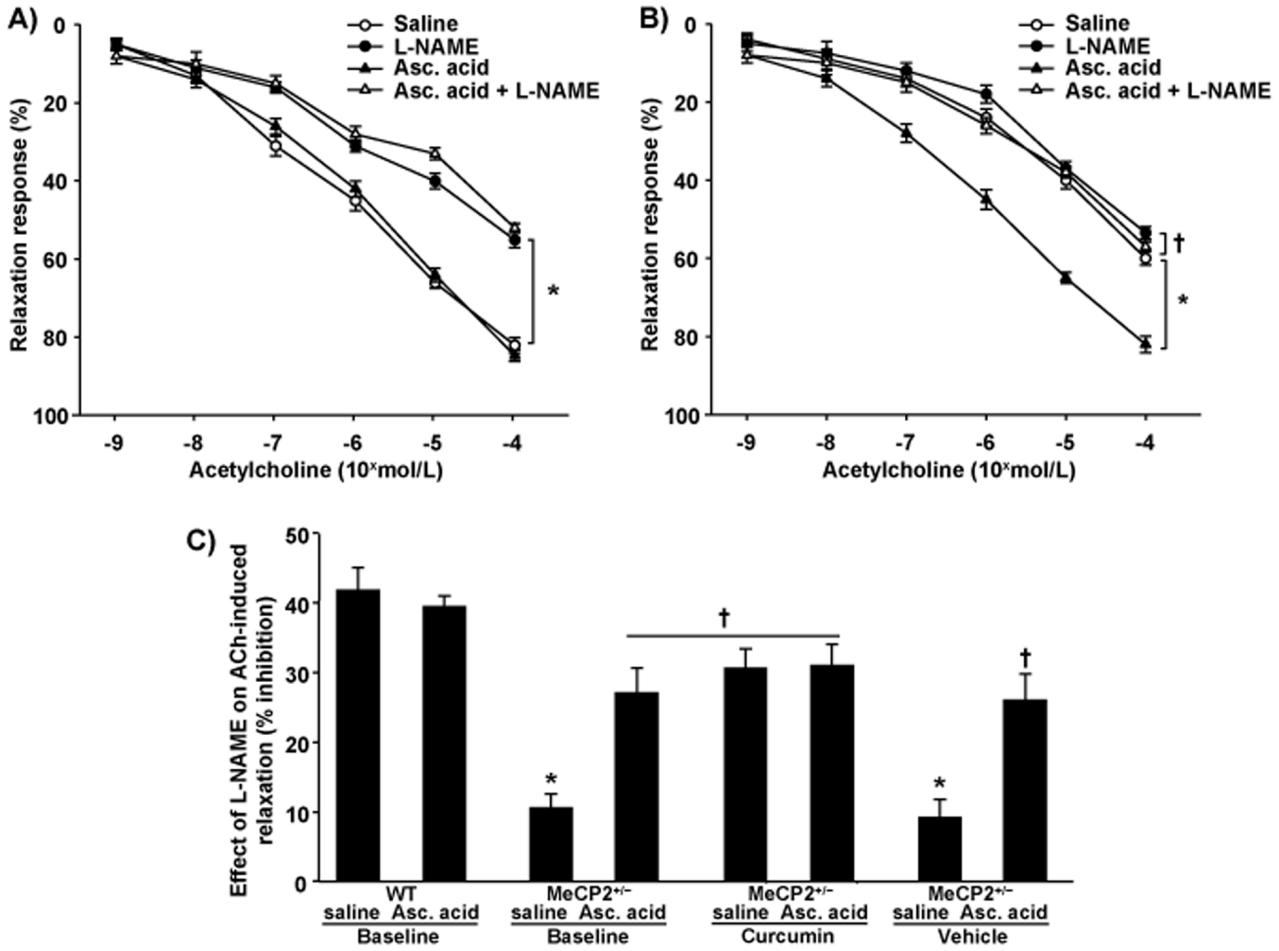
Endothelial relaxation in curcumin or vehicle-treated mice. Relaxations to acetylcholine without (saline) or with L-NAME, ascorbic acid (Asc. acid), or both, in curcumin-treated (panel A) and not simultaneously vehicle-treated (panel B) MeCP2^+/−^ female mice. *P<0.001 †*P*<0.001. Panel C: Bar graph indicates the inhibition by L-NAME on maximal response to acetylcholine (ACh) in vessels from WT and MeCP2^+/−^ at baseline and in vessels from MeCP2^+/−^ mice after curcumin or vehicle administration, without (saline) or with ascorbic (asc) acid. *P<0.001 vs WT, †*P*<0.05 vs MeCP2^+/−^ saline at baseline. Results are given as mean±SEM.

In mesenteric vessels from the WT group (n = 9), the relaxation to acetylcholine was preserved, significantly attenuated by L-NAME and not affected by ascorbic acid (Figures 1A and 2B). Vessels from MeCP2^+/−^ female mice showed a significant (2-way ANOVA, comparison between curves in panels A and C of Figure 1, *P*<0.001) attenuated relaxation to acetylcholine as compared to WT controls. Indeed, in this group, the inhibitory effect of L-NAME on relaxation to acetylcholine, although still evident at the maximal dose, was significantly blunted. In addition, ascorbic acid potentiated the relaxation to acetylcholine and restored the inhibitory effect of L-NAME (Figures 1C and 2C). Similar results were obtained on a group of MeCP2^+/−^ female mice bred on the pure 129Sv background (see Figure S1A)

Vessels from MeCP2^−/y^ male mice (Figure 1D) were characterized by an exacerbated reduced relaxation to acetylcholine (with respect to relative WT, Figure 1B) as compared to MeCP2^+/−^ female mice (2-way ANOVA, comparison between curves in panels D and C of Figure 1, *P*<0.05), which was totally resistant to L-NAME. Ascorbic acid enhanced only in part the relaxation to acetylcholine and restored the inhibition by L-NAME.

Similarly to results obtained with acetylcholine, endothelium-dependent relaxation to bradykinin was significantly blunted in vessels from MeCP2^+/−^ female mice compared with controls (E_max_: 59.6±1.8% vs 97.1±1.2%, respectively; *P*<0.001), and additionally attenuated in MeCP2^y/−^ male mice (E_max_: 48.2±1.4%; *P*<0.01 vs MeCP2^+/−^).

Relaxation to sodium nitroprusside was similar in female and male WT and MeCP2^+/−^ animals (E_max_: 97.6±1.3% and 97.5±1.1%, respectively), but it was significantly attenuated in MeCP2^−/y^ mice (E_max_: 80.9±1.4%; *P*<0.01 vs other groups).

### Effect of curcumin administration on endothelial function

To evaluate how an antioxidant treatment impacts on the endothelial phenotype described above symptomatic MeCP2 ^+/−^ and WT female mice were treated with curcumin for 3 weeks. Mice were fed with food containing curcumin at 5% weight/weight, a dose proved to result in micromolar concentration of plasmatic curcumin [43]. Indeed plasma concentration of curcumin, as assessed by HPLC, was 20.6 µg/ml ±1.6 and 19.75 µg/ml ±1.4 in WT and MeCP2 +/− mice respectively.

**Figure 3:**
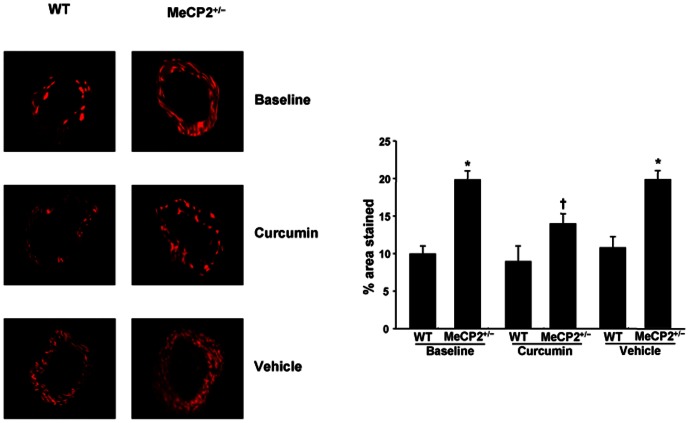
Superoxide anion generation: DHE staining and quantification. Representative DHE staining and quantification (bar graph) of the red signal in mesenteric arteries (magnification X 40) from WT and MeCP2^+/−^ mice at baseline and after curcumin or vehicle (not simultaneous to curcumin) administration. Results for 6 experiments are given as mean±SEM. *P<0.01 vs WT, †*P*<0.05 vs MeCP2^+/−^ at baseline.

**Figure 4:**
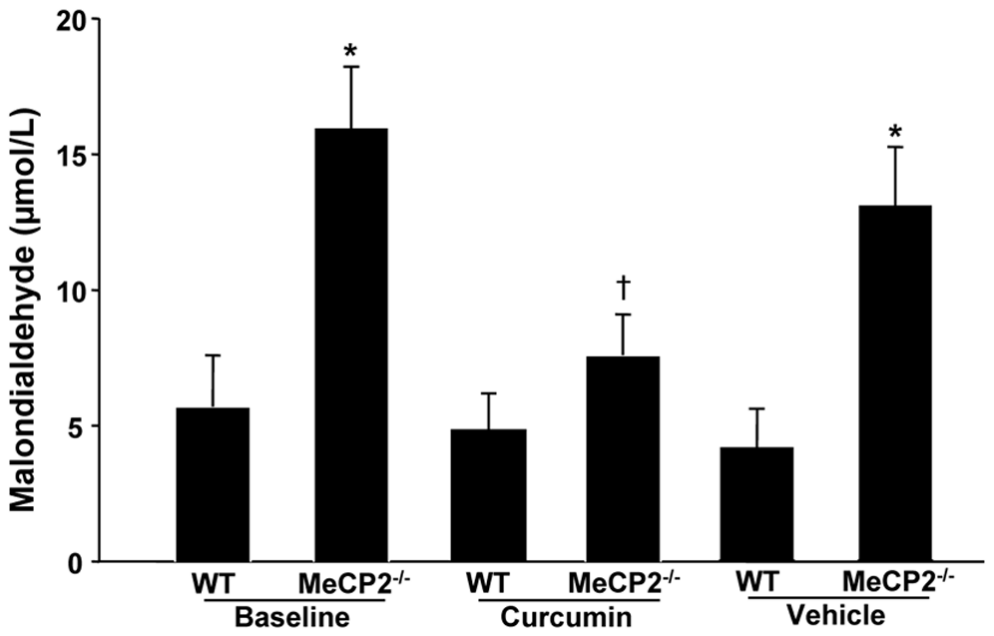
Malondialdehyde (MDA) levels. Plasma levels of MDA of WT and MeCP2^+/−^ mice at baseline and after treatment with curcumin or vehicle (not simultaneous) *P<0.01 vs other groups; †*P*<0.05 vs WT.

**Figure 5:**
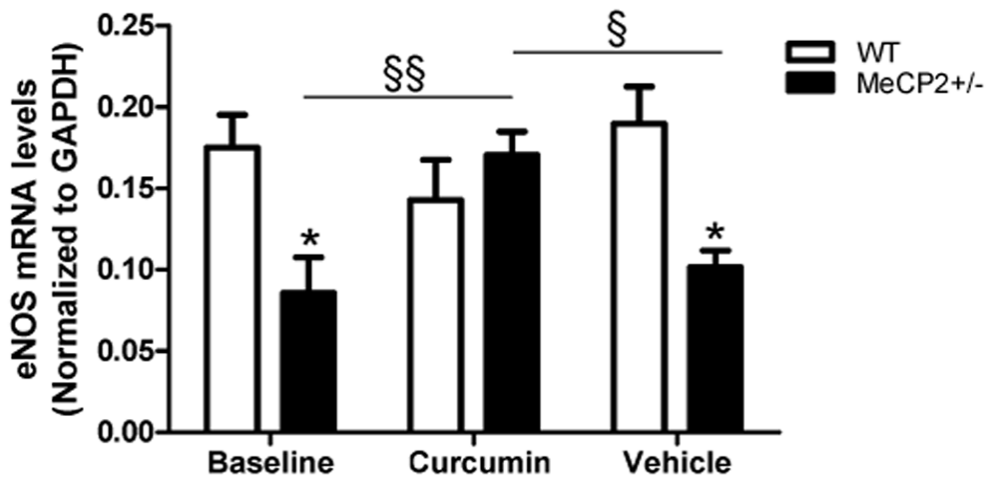
eNOS mRNA expression. Quantitative RT-PCR analysis of eNOS mRNA expression in mesenteric vessels from WT and MeCP2^+/−^ mice at baseline and after treatment with curcumin or vehicle (not simultaneous). eNOS has been normalized to the expression of GAPDH and data are delta CT-transformations from real time plots 2-way ANOVA: Genotype effect P<0.05, interaction P<0.01, Bonferroni Post hoc tests: *P<0.01 vs WT; § *P*<0.05 §§P<0.01 vs MeCP2^+/−^ curcumin treated.

Food consumption was similar in all groups analyzed: neither the comparison between curcumin WT versus curcumin RTT (6.3±0.7 and 7.3±0.6 g respectively), nor the comparison between untreated (referred to as baseline in Figures 2–5) and curcumin treated RTT animals (8.6±0.4 and 7.3±0.6g respectively) revealed any significant difference. The treatment dramatically potentiated (2-way ANOVA, curves in panels 1C and 2B, *P*<0.001), but not normalized (comparison Figures 1A and 2B, *P*<0.05), the relaxation to acetylcholine in mesenteric vessels from MeCP2^+/−^ mice, and restored the inhibitory effect of L-NAME on acetylcholine-induced relaxation (Figures 2B and 2C). In such arteries, the vascular response to acetylcholine was no longer affected by ascorbic acid (Figure 2B). Similar results were obtained on a group of MeCP2^+/−^ female mice bred on the pure 129Sv background (see Figure S1B). On the contrary, in vessels from WT mice, curcumin administration did not modify the relaxation to acetylcholine (E_max_: 97.5±0.9%), the null effect of ascorbic acid (E_max_: 96.8±0.5%), 96.4±0.6%), or the inhibitory effect of L-NAME on acetylcholine (E_max_: 58.7±1.2%; inhibition: −38.1±0.6%), (2-way ANOVA, comparison between female WT baseline and female WT curcumin *P*<0.05)

Relaxations to sodium nitroprusside were not modified by curcumin administration in MeCP2^+/−^ and WT (E_max_: 97.5±0.5% and 96.9±1.1%, respectively).

### Time-control study

To evaluate if and how the time employed for curcumin treatment (3 weeks) impacted on the natural progression of the disease in terms of endothelial function, separate and not simultaneously analyzed groups of WT and symptomatically matched MeCP2^+/−^ female mice were treated with placebo for 3 weeks. Mice were fed with food prepared as described for the curcumin added food, but not containing the drug (this group will be referred to as “Vehicle”). In vessels from MeCP2^+/−^ female mice, vehicle administration did not significantly modify the relaxation to acetylcholine, the slight inhibition by L-NAME on acetylcholine, or the potentiating effect of ascorbic acid, which also restored the inhibitory effect of L-NAME on acetylcholine (Figures 2A, 2C). Similarly, in vessels from WT female mice, 3 weeks of vehicle treatment failed to affect the preserved vascular response to acetylcholine (E_max_: 95.8±0.6%), the null effect of ascorbic acid (E_max_: 96.1±0.8%), or the inhibitory effect of L-NAME on acetylcholine (E_max_: 57.3±1.5%; inhibition: −38.5±0.9%).

Relaxations to sodium nitroprusside were not modified during the 3 wks period period in MeCP2^+/−^ and WT groups (E_max_: 97.3±0.8% and 96.9±1.6%, respectively) with respect to baseline.

### Effects of curcumin administration on vascular superoxide generation and malondialdehyde levels

In small arteries from MeCP2^+/−^ mice, DHE analysis revealed a dramatic increase in superoxide anion production, as compared with WT (Figure 3). The enhanced superoxide generation was significantly attenuated by curcumin but not affected by vehicle treatment (Figure 3). In vessels from WT, neither curcumin nor vehicle administration significantly modified the superoxide detection (Figure 3). In control experiments no autofluorescence for elastin emerged (data not shown). MeCP2^+/−^ animals showed also higher plasma values of malondialdehyde, which were significantly attenuated, but not normalized, by curcumin and not significantly modified by vehicle (Figure 4).

### Vascular eNOS expressions

eNOS mRNA expression was measured in mesenteric vessels of Rett animals and WT age-matched littermates. The eNOS mRNA expression from the mesenteric arterioles was significantly decreased in MeCP2^+/−^ mice respect to WT age-matched littermates, an effect totally reversed by curcumin treatment, but not modified by vehicle intake. Of note, in the presence of curcumin, vascular eNOS expression was no longer different from that of WT animals (Figure 5).

### Vascular eNOS immunostaining

**Figure 6:**
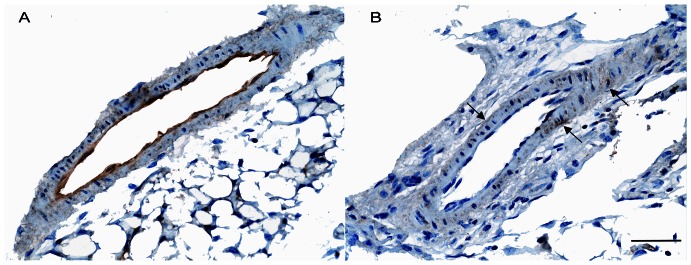
eNOS immunostaining. Representative photomicrograph of immunostaining for eNOS in small mesenteric arteries from WT (A) and MeCP2^+/−^ mice (B). A strong, specific eNOS staining is detected in endothelium of control vessels (A), while mesenteric arterioles from MeCP2^+/−^ mice show only a slight eNOS immunostaining corresponding to the outer vascular smooth muscle cells, without any appreciable amount in the endothelial cell layer (B, arrows). Scale bar 50 µm.

In small mesenteric arterioles from WT mice, eNOS was constitutively expressed within endothelial cells at 1.94% of the immunoreactive area (SD±0.44) (Figure 6A). In contrast, vessels from MeCP2^+/−^ animals displayed a significant reduction of (*P*<0.001) eNOS immunostaining down to 0.035% of the immunoreactive area (SD±0.01) of the outer vascular smooth muscle cells, without any appreciable amount at the endothelial level (Figure 6B).

## Discussion

In this study we investigated for the first time the function of the peripheral microcirculation in a symptomatic MeCP2 knockout mouse model, and the impact of curcumin administration on its vascular function. Our findings indicate that these animals are characterized by an altered endothelium-dependent relaxation caused by a reduced NO availability due to an increased intravascular ROS generation. Interestingly, means of curcumin treatment. we were able to rescue the vasculature dysfunction

The prominent CNS effects of the RTT syndrome have so far directed a major research effort into the investigation of alterations taking place in the central nervous system, almost neglecting the other somatic peripheral symptoms [22], including the circulatory problems often taking place during the first year of life of RTT patients [51].

The first finding of the present study concerns the demonstration of endothelial dysfunction and the underlying mechanisms responsible for this alteration in RTT male (MeCP2^−/y^) and female (MeCP2^+/−^) animal model. Specifically, in the mesenteric arterioles of the MeCP2^+/−^ female mice, we observed a dramatic reduction in the vascular response to acetylcholine, an endothelium-dependent vasodilator, together with a preserved relaxation to sodium nitroprusside, a direct smooth muscle cell relaxant compound, indicating the presence of endothelial dysfunction. Of note, the reduced endothelial function observed in our MeCP2^+/−^ animal model was obtained with both acetylcholine and bradykinin. These 2 agonists act on different receptors and G protein dependent signal transduction that are respectively sensitive and insensitive to pertussis toxin [48]. Therefore, our findings indicate that endothelial dysfunction is not related to a specific defect of the muscarinic receptor for acetylcholine or to an abnormality of a single intracellular signal-transduction pathway, but to a more generalized abnormality of endothelial vasodilator function present in this animal model.

NO availability and oxidative stress, key parameters of endothelial functionality, were specifically measured through the selective NOS inhibitor L-NAME and the ROS scavenger ascorbic acid, respectively. In MeCP2^+/−^ mice, the inhibitory effect exerted by L-NAME on acetylcholine responses was lower compared with that observed in controls, indicating reduced NO availability. Ascorbic acid improved the response to acetylcholine in RTT mice, suggesting that ROS generation contributes to the endothelial dysfunction observed in this model. When L-NAME was retested simultaneously with ascorbic acid, its inhibitory effect on acetylcholine was restored, indicating that in the presence of ROS scavenging, the activity of the L-arginine-NO pathway was re-established. Taken together, these findings indicate that ROS generation represents a major mechanism accounting for endothelial dysfunction in peripheral microcirculation of MeCP2^+/−^ mice, resulting in NO breakdown. In line with functional data, MeCP2^+/−^ mice showed an increased intravascular superoxide anion generation. The oxidant excess in these animals was also confirmed by higher plasma values of MDA, a systemic marker of oxidative stress. Of note, MeCP2^−/y^ male animals showed a more marked endothelial dysfunction compared with MeCP2^+/−^ female mice, suggesting that the mosaicism of the female animals is sufficient to partially maintain the functional activity of the vessels. Indeed, in the vessels of male mice the NO availability is almost absent, as evidenced by the acetylcholine-induced relaxation,totally refractory to L-NAME, while ascorbic acid only slightly restored the inhibitory effect of L-NAME, suggesting a quite irreversible exhaustion of the L-arginine-NO pathway. In such context a reduced vascular response to sodium nitroprusside also emerged, indicating that the detrimental effect of MECP2 deletion on the vasculature is no longer limited to endothelial function but it involves the underlying smooth muscle cells. The central nervous system is particularly sensitive to the adverse effects of oxidative stress [52] and the presence of systemic oxidative stress in RTT patients constitutes an intriguing and long debated aspect in Rett syndrome pathogenesis [53]. In this perspective, our findings in rodents are in line with the elevated plasma levels of oxidative stress, decreased superoxide dismutase activity, and increased markers of lipid peroxidation [53], [54] reported in MeCP2-RTT patients.

Vessels from curcumin-treated MeCP2^+/−^ mice showed an increased relaxation to acetylcholine, which became sensitive to L-NAME, while ascorbic acid was no longer able to affect the response to acetylcholine. The demonstration that in the presence of curcumin L-NAME can inhibit relaxation to acetylcholine, an effect not exerted under baseline conditions, implies that NO availability is restored after treatment. It is worth noticing that after curcumin administration the restored inhibiting effect of L-NAME on acetylcholine was similar to what observed upon acute ascorbic acid treatment at baseline. Therefore these findings suggest that the mechanism responsible for the beneficial effect of curcumin is probably specific, and related to its antioxidant properties. This concept is strengthened by curcumin capacity to attenuate the intravascular superoxide excess, and to reduce the increased plasma levels of MDA. Of note, the lack of any significant impact by vehicle intake on endothelial dysfunction in MeCP2^+/−^ mice rules out any active role played by the natural progression of the disease and the observed beneficial effects can thus be attributed to the pharmacological treatment, at least in the specific time period investigated.

Our findings extend to the peripheral microcirculation of MeCP2^+/−^ mice previous evidence showing the beneficial antioxidant properties of curcumin on other animal models affected by oxidative damage [55], [56]. It is worth observing that in the presence of ascorbic acid, both the relaxing effect of acetylcholine and the inhibitory effect of L-NAME on acetylcholine, although significantly improved, did not reach normal values. Thus, we cannot exclude that other ROS-independent mechanisms may contribute to the MeCP2^+/−^-related endothelial dysfunction. Likewise, we recognize that the DHE analysis utilized in the present study does not allow to exclude that other ROS different from superoxide may participate in generating intravascular oxidative stress.

Previous reports documented the selective presence of MeCP2 at promoter level of eNOS [28], [29], thus raising the hypothesis that the MECP2 mutation might extend its negative influence to the regulation of vascular eNOS, the major putative intravascular source of NO. In line with this concept and with the reduced NO availability herein reported, isolated mesenteric vessels of MeCP2 deficient mice exhibited decreased eNOS mRNA expression and marked reduction of eNOS immunostaining, mainly at the level of endothelial layer, thus providing a mechanistic, although indirect, insight able to link MECP2 mutation with reduced vascular eNOS expression in RTT animals. When considering that intravascular ROS generation is the major mechanism accounting for the NO breakdown, we can hypothesize that the reduced NO availability might represent a stimulus for endothelial cells to generate eNOS, with the consequent inevitable exhaustion of the enzyme. In such context, the lower eNOS expression may be considered as the exhausted compensatory attempt by endothelium to generate NO. Accordingly, chronic antioxidant curcumin might allow the endothelial cells to restore an adequate availability of the enzymatic pathway to generate NO. Alternatively, eNOS down-regulation and ROS excess might represent two simultaneous alterations which, although generated independently, act synergistically to worse NO availability. This issue needs future investigations.

It is widely recognized that in physiological conditions, vascular NO not only causes vasodilation, but also protects the vessel wall against the development of atherosclerosis [57]. In contrast, the impaired NO activity and the enhanced activity of vasoconstrictors, including ROS, are responsible for impaired vasodilator capacity. The imbalance of this equilibrium towards oxidative stress, beyond the altered vasomotricity, also initiates a variety of active processes, such as inflammation, cell migration and proliferation, relevant for the endothelial surface and adjacent wall tissues. For these reasons the altered NO-mediated endothelial function and the increased ROS generation which characterize the peripheral microcirculation of MeCP2 knockout animals, might have a causal relationship with the poor circulation present in the extremities of RTT patients, including cold and cyanotic legs.

In conclusion, the present study indicates that mesenteric vessels from MeCP2^+/−^ female mice are characterized by an altered endothelium-dependent relaxation caused by a reduced NO availability due to an increased ROS generation, and by a lower vascular eNOS expression. These alterations, as well as the pathological stereotyped movements that characterize the pathological animal, were partially reversed by curcumin administration. These data revealed that the RTT animal model shares some of the components of the clinical oxidative and vascular impairment of RTT patients, who often present circulatory problems that become increasingly severe in adulthood. It is proposed that the peripheral vascular system of these animals may be a source of excessive free radicals contributing to the local and systemic oxidative stress, and that curcumin based therapy may at least partly improve RTT symptoms derived from these redox alterations.

## Supporting Information

Figure S1 **Endothelial relaxation in pure 129SV MeCP2^+/−^ mice and effect of curcumin treatment.** Endothelium-dependent relaxations elicited by Acetylcholine without (saline) or with L-NAME, ascorbic acid or both, in mesenteric resistance arteries from MeCP2^+/−^ female mice at baseline (A), or after curcumin treatment (B). Each point represents the mean of 4 animals±SEM. *P<0.001, **†** *P*<0.05. Mean body weight was 18.9±1.6 g and 18.9±0.86 g for untreated and curcumin treated mice respectively
(TIF)

Table S1 Physical parameters of mice employed in the curcumin experiment.
(DOC)

## References

[1] Amir RE, Van den Veyver IB, Wan M, Tran CQ, Francke U, et al (1999) Rett syndrome is caused by mutations in X-linked MECP2, encoding methyl-CpG-binding protein 2. Nat Genet 23: 185–188. 10508514 10.1038/13810

[2] Chahrour M, Zoghbi HY (2007) The story of Rett syndrome: from clinic to neurobiology. Neuron 56: 422–437. 17988628 10.1016/j.neuron.2007.10.001

[3] Lasalle JM, Yasui DH (2009) Evolving role of MeCP2 in Rett syndrome and autism. Epigenomics 1: 119–130. 20473347 10.2217/epi.09.13 PMC2867478

[4] Neul JL, Kaufmann WE, Glaze DG, Christodoulou J, Clarke AJ, et al (2010) Rett syndrome: revised diagnostic criteria and nomenclature. Ann Neurol 68: 944–950. 21154482 10.1002/ana.22124 PMC3058521

[5] Shahbazian MD, Zoghbi HY (2001) Molecular genetics of Rett syndrome and clinical spectrum of MECP2 mutations. Curr Opin Neurol 14: 171–176. 11262731 10.1097/00019052-200104000-00006

[6] Neul JL, Fang P, Barrish J, Lane J, Caeg EB, et al (2008) Specific mutations in methyl-CpG-binding protein 2 confer different severity in Rett syndrome. Neurology 70: 1313–1321. 18337588 10.1212/01.wnl.0000291011.54508.aa PMC2677974

[7] Caballero IM, Hendrich B (2005) MeCP2 in neurons: closing in on the causes of Rett syndrome. Hum Mol Genet 14 Spec No 1: R19–26. 10.1093/hmg/ddi102 15809268

[8] Moretti P, Zoghbi HY (2006) MeCP2 dysfunction in Rett syndrome and related disorders. Curr Opin Genet Dev 16: 276–281. 16647848 10.1016/j.gde.2006.04.009

[9] Smeets EE, Pelc K, Dan B (2012) Rett Syndrome. Mol Syndromol 2: 113–127. 22670134 10.1159/000337637 PMC3366703

[10] Glaze DG (2004) Rett syndrome: of girls and mice-lessons for regression in autism. Ment Retard Dev Disabil Res Rev 10: 154–158. 15362175 10.1002/mrdd.20030

[11] Lappalainen R, Liewendahl K, Sainio K, Nikkinen P, Riikonen RS (1997) Brain perfusion SPECT and EEG findings in Rett syndrome. Acta Neurol Scand 95: 44–50. 9048985 10.1111/j.1600-0404.1997.tb00067.x

[12] Bianciardi G, Acampa M, Lamberti I, Sartini S, Servi M, et al.. (2013) Microvascular abnormalities in Rett syndrome. Clin Hemorheol Microcirc. 10.3233/CH-131707 23481597

[13] Bumin G, Uyanik M, Yilmaz I, Kayihan H, Topcu M (2003) Hydrotherapy for Rett syndrome. J Rehabil Med 35: 44–45. 12610848 10.1080/16501970306107

[14] Guy J, Hendrich B, Holmes M, Martin JE, Bird A (2001) A mouse Mecp2-null mutation causes neurological symptoms that mimic Rett syndrome. Nat Genet 27: 322–326. 11242117 10.1038/85899

[15] Chen RZ, Akbarian S, Tudor M, Jaenisch R (2001) Deficiency of methyl-CpG binding protein-2 in CNS neurons results in a Rett-like phenotype in mice. Nat Genet 27: 327–331. 11242118 10.1038/85906

[16] Pelka GJ, Watson CM, Radziewic T, Hayward M, Lahooti H, et al (2006) Mecp2 deficiency is associated with learning and cognitive deficits and altered gene activity in the hippocampal region of mice. Brain 129: 887–898. 16467389 10.1093/brain/awl022

[17] Shahbazian M, Young J, Yuva-Paylor L, Spencer C, Antalffy B, et al (2002) Mice with truncated MeCP2 recapitulate many Rett syndrome features and display hyperacetylation of histone H3. Neuron 35: 243–254. 12160743 10.1016/s0896-6273(02)00768-7

[18] Katz DM, Berger-Sweeney JE, Eubanks JH, Justice MJ, Neul JL, et al (2012) Preclinical research in Rett syndrome: setting the foundation for translational success. Dis Model Mech 5: 733–745. 23115203 10.1242/dmm.011007 PMC3484856

[19] Percy AK (2011) Rett syndrome: exploring the autism link. Arch Neurol 68: 985–989. 21825235 10.1001/archneurol.2011.149 PMC3674963

[20] O'Connor RD, Zayzafoon M, Farach-Carson MC, Schanen NC (2009) Mecp2 deficiency decreases bone formation and reduces bone volume in a rodent model of Rett syndrome. Bone 45: 346–356. 19414073 10.1016/j.bone.2009.04.251 PMC2739100

[21] Moretti P, Levenson JM, Battaglia F, Atkinson R, Teague R, et al (2006) Learning and memory and synaptic plasticity are impaired in a mouse model of Rett syndrome. J Neurosci 26: 319–327. 16399702 10.1523/JNEUROSCI.2623-05.2006 PMC6674314

[22] Stearns NA, Schaevitz LR, Bowling H, Nag N, Berger UV, et al (2007) Behavioral and anatomical abnormalities in Mecp2 mutant mice: a model for Rett syndrome. Neuroscience 146: 907–921. 17383101 10.1016/j.neuroscience.2007.02.009

[23] Pearson BL, Defensor EB, Pobbe RL, Yamamoto LH, Bolivar VJ, et al (2012) Mecp2 truncation in male mice promotes affiliative social behavior. Behav Genet 42: 299–312. 21909962 10.1007/s10519-011-9501-2 PMC5843946

[24] Bissonnette JM, Knopp SJ, Maylie J, Thong T (2007) Autonomic cardiovascular control in methyl-CpG-binding protein 2 (Mecp2) deficient mice. Auton Neurosci 136: 82–89. 17544925 10.1016/j.autneu.2007.04.007 PMC2866300

[25] McCauley MD, Wang T, Mike E, Herrera J, Beavers DL, et al (2011) Pathogenesis of lethal cardiac arrhythmias in Mecp2 mutant mice: implication for therapy in Rett syndrome. Sci Transl Med 3: 113ra125. 10.1126/scitranslmed.3002982 PMC3633081 22174313

[26] Luscher TF, Barton M (1997) Biology of the endothelium. Clin Cardiol 20: II–3-10. 10.1002/j.1932-8737.1997.tb00006.x PMC7509692 9422846

[27] Vanhoutte PM, Mombouli JV (1996) Vascular endothelium: vasoactive mediators. Prog Cardiovasc Dis 39: 229–238. 8970575 10.1016/s0033-0620(96)80003-x

[28] Chan Y, Fish JE, D'Abreo C, Lin S, Robb GB, et al (2004) The cell-specific expression of endothelial nitric-oxide synthase: a role for DNA methylation. J Biol Chem 279: 35087–35100. 15180995 10.1074/jbc.M405063200

[29] Fish JE, Matouk CC, Rachlis A, Lin S, Tai SC, et al (2005) The expression of endothelial nitric-oxide synthase is controlled by a cell-specific histone code. J Biol Chem 280: 24824–24838. 15870070 10.1074/jbc.M502115200

[30] Wu A, Ying Z, Gomez-Pinilla F (2006) Dietary curcumin counteracts the outcome of traumatic brain injury on oxidative stress, synaptic plasticity, and cognition. Exp Neurol 197: 309–317. 16364299 10.1016/j.expneurol.2005.09.004

[31] Aggarwal BB, Harikumar KB (2009) Potential therapeutic effects of curcumin, the anti-inflammatory agent, against neurodegenerative, cardiovascular, pulmonary, metabolic, autoimmune and neoplastic diseases. Int J Biochem Cell Biol 41: 40–59. 18662800 10.1016/j.biocel.2008.06.010 PMC2637808

[32] Wang R, Li YB, Li YH, Xu Y, Wu HL, et al (2008) Curcumin protects against glutamate excitotoxicity in rat cerebral cortical neurons by increasing brain-derived neurotrophic factor level and activating TrkB. Brain Res 1210: 84–91. 18420184 10.1016/j.brainres.2008.01.104

[33] Ataie A, Sabetkasaei M, Haghparast A, Moghaddam AH, Kazeminejad B (2010) Neuroprotective effects of the polyphenolic antioxidant agent, Curcumin, against homocysteine-induced cognitive impairment and oxidative stress in the rat. Pharmacol Biochem Behav 96: 378–385. 20619287 10.1016/j.pbb.2010.06.009

[34] Garcia-Alloza M, Borrelli LA, Rozkalne A, Hyman BT, Bacskai BJ (2007) Curcumin labels amyloid pathology in vivo, disrupts existing plaques, and partially restores distorted neurites in an Alzheimer mouse model. J Neurochem 102: 1095–1104. 17472706 10.1111/j.1471-4159.2007.04613.x

[35] Kim DS, Park SY, Kim JK (2001) Curcuminoids from Curcuma longa L. (Zingiberaceae) that protect PC12 rat pheochromocytoma and normal human umbilical vein endothelial cells from betaA(1–42) insult. Neurosci Lett 303: 57–61. 11297823 10.1016/s0304-3940(01)01677-9

[36] Bora-Tatar G, Dayangac-Erden D, Demir AS, Dalkara S, Yelekci K, et al (2009) Molecular modifications on carboxylic acid derivatives as potent histone deacetylase inhibitors: Activity and docking studies. Bioorg Med Chem 17: 5219–5228. 19520580 10.1016/j.bmc.2009.05.042

[37] Chen Y, Shu W, Chen W, Wu Q, Liu H, et al (2007) Curcumin, both histone deacetylase and p300/CBP-specific inhibitor, represses the activity of nuclear factor kappa B and Notch 1 in Raji cells. Basic Clin Pharmacol Toxicol 101: 427–433. 17927689 10.1111/j.1742-7843.2007.00142.x

[38] Gonzales ML, LaSalle JM (2010) The role of MeCP2 in brain development and neurodevelopmental disorders. Curr Psychiatry Rep 12: 127–134. 20425298 10.1007/s11920-010-0097-7 PMC2847695

[39] Boggio EM, Lonetti G, Pizzorusso T, Giustetto M (2010) Synaptic determinants of rett syndrome. Front Synaptic Neurosci 2: 28. 21423514 10.3389/fnsyn.2010.00028 PMC3059682

[40] Lonetti G, Angelucci A, Morando L, Boggio EM, Giustetto M, et al (2010) Early environmental enrichment moderates the behavioral and synaptic phenotype of MeCP2 null mice. Biol Psychiatry 67: 657–665. 20172507 10.1016/j.biopsych.2009.12.022

[41] Ricciardi S, Boggio EM, Grosso S, Lonetti G, Forlani G, et al (2011) Reduced AKT/mTOR signaling and protein synthesis dysregulation in a Rett syndrome animal model. Hum Mol Genet 20: 1182–1196. 21212100 10.1093/hmg/ddq563

[42] Coban D, Milenkovic D, Chanet A, Khallou-Laschet J, Sabbe L, et al (2012) Dietary curcumin inhibits atherosclerosis by affecting the expression of genes involved in leukocyte adhesion and transendothelial migration. Mol Nutr Food Res 56: 1270–1281. 22753158 10.1002/mnfr.201100818

[43] Lee JC, Kinniry PA, Arguiri E, Serota M, Kanterakis S, et al (2010) Dietary curcumin increases antioxidant defenses in lung, ameliorates radiation-induced pulmonary fibrosis, and improves survival in mice. Radiat Res 173: 590–601. 20426658 10.1667/RR1522.1 PMC2873679

[44] Sharma RA, Gescher AJ, Steward WP (2005) Curcumin: the story so far. Eur J Cancer 41: 1955–1968. 16081279 10.1016/j.ejca.2005.05.009

[45] Li J, Jiang Y, Wen J, Fan G, Wu Y, et al (2009) A rapid and simple HPLC method for the determination of curcumin in rat plasma: assay development, validation and application to a pharmacokinetic study of curcumin liposome. Biomed Chromatogr 23: 1201–1207. 19488971 10.1002/bmc.1244

[46] Virdis A, Colucci R, Fornai M, Duranti E, Giannarelli C, et al (2007) Cyclooxygenase-1 is involved in endothelial dysfunction of mesenteric small arteries from angiotensin II-infused mice. Hypertension 49: 679–686. 17145980 10.1161/01.HYP.0000253085.56217.11

[47] Flammer AJ, Anderson T, Celermajer DS, Creager MA, Deanfield J, et al (2012) The assessment of endothelial function: from research into clinical practice. Circulation 126: 753–767. 22869857 10.1161/CIRCULATIONAHA.112.093245 PMC3427943

[48] Flavahan NA, Shimokawa H, Vanhoutte PM (1991) Inhibition of endothelium-dependent relaxations by phorbol myristate acetate in canine coronary arteries: role of a pertussis toxin-sensitive G-protein. J Pharmacol Exp Ther 256: 50–55. 1899121

[49] Virdis A, Iglarz M, Neves MF, Amiri F, Touyz RM, et al.. (2003) Effect of hyperhomocystinemia and hypertension on endothelial function in methylenetetrahydrofolate reductase-deficient mice. Arterioscler Thromb Vasc Biol 23: 1352–1357. Epub 2003 Jun 1326. 10.1161/01.ATV.0000083297.47245.DA 12829522

[50] Virdis A, Santini F, Colucci R, Duranti E, Salvetti G, et al (2011) Vascular generation of tumor necrosis factor-alpha reduces nitric oxide availability in small arteries from visceral fat of obese patients. J Am Coll Cardiol 58: 238–247. 21737013 10.1016/j.jacc.2011.01.050

[51] Bjure J, Uvebrant P, Vestergren E, Hagberg B (1997) Regional cerebral blood flow abnormalities in Rett syndrome. Eur Child Adolesc Psychiatry 6 Suppl 164–66. 9452923

[52] Atmaca M, Tezcan E, Kuloglu M, Ustundag B, Tunckol H (2004) Antioxidant enzyme and malondialdehyde values in social phobia before and after citalopram treatment. Eur Arch Psychiatry Clin Neurosci 254: 231–235. 15309392 10.1007/s00406-004-0484-3

[53] De Felice C, Ciccoli L, Leoncini S, Signorini C, Rossi M, et al (2009) Systemic oxidative stress in classic Rett syndrome. Free Radic Biol Med 47: 440–448. 19464363 10.1016/j.freeradbiomed.2009.05.016

[54] Sierra C, Vilaseca MA, Brandi N, Artuch R, Mira A, et al (2001) Oxidative stress in Rett syndrome. Brain Dev 23 Suppl 1S236–239. 11738881 10.1016/s0387-7604(01)00369-2

[55] Majithiya JB, Balaraman R (2005) Time-dependent changes in antioxidant enzymes and vascular reactivity of aorta in streptozotocin-induced diabetic rats treated with curcumin. J Cardiovasc Pharmacol 46: 697–705. 16220078 10.1097/01.fjc.0000183720.85014.24

[56] Sompamit K, Kukongviriyapan U, Nakmareong S, Pannangpetch P, Kukongviriyapan V (2009) Curcumin improves vascular function and alleviates oxidative stress in non-lethal lipopolysaccharide-induced endotoxaemia in mice. Eur J Pharmacol 616: 192–199. 19540224 10.1016/j.ejphar.2009.06.014

[57] Davignon J, Ganz P (2004) Role of endothelial dysfunction in atherosclerosis. Circulation 109: III27–32. 15198963 10.1161/01.CIR.0000131515.03336.f8

