# Supplementary figures and images for: Vascular Dysfunction in a Mouse Model of Rett Syndrome and Effects of Curcumin Treatment
Source: PLoS One. 2013 May 21;8(5):e64863. doi: 10.1371/journal.pone.0064863 (PMC3660336; doi:10.1371/journal.pone.0064863)

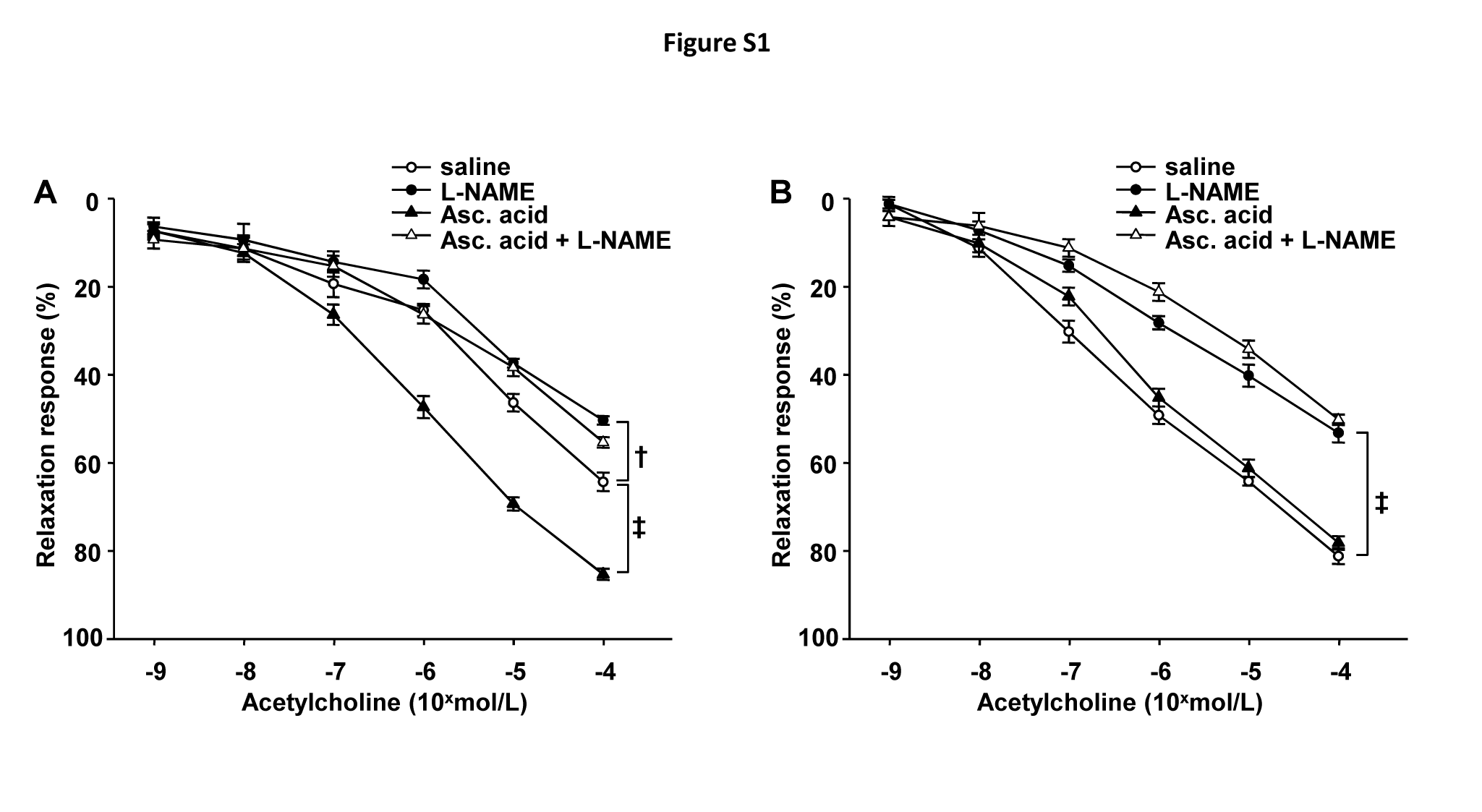

Supplement: Figure S1 — Endothelial relaxation in pure 129SV MeCP2+/− mice and effect of curcumin treatment. Endothelium-dependent relaxations elicited by Acetylcholine without (saline) or with L-NAME, ascorbic acid or both, in mesenteric resistance arteries from MeCP2+/− female mice at baseline (A), or after curcumin treatment (B). Each point represents the mean of 4 animals±SEM. *P<0.001, † P<0.05. Mean body weight was 18.9±1.6 g and 18.9±0.86 g for untreated and curcumin treated mice respectively (TIF) [file pone.0064863.s001.tif]
