# Supplementary material for: Vascular Dysfunction in a Mouse Model of Rett Syndrome and Effects of Curcumin Treatment
Source: PLoS One. 2013 May 21;8(5):e64863. doi: 10.1371/journal.pone.0064863 (PMC3660336; doi:10.1371/journal.pone.0064863)
Supplement: Table S1 — Physical parameters of mice employed in the curcumin experiment. (DOC) [file pone.0064863.s002.doc]

**Table S1.** Physical parameters of mice employed in the curcumin experiment

**Table S1**. Physical parameters of mice employed in the curcumin experiment

|  | WT untreated  n=9 | MeCP2^+/-^ untreated  n=9 | WT Curcumin  n=9 | MeCP2^+/-^ Curcumin  n=9 |
| --- | --- | --- | --- | --- |
| Age (wks) | 24±1.0 | 25±0.9 | 25±1.1 | 24±0.7 |
| Body weight (g)  Day 0 | 23.14±1.12 | 19.58±0.92 | 23.0±1.27 | 21.11±1.37 |
| Weight gain (g) | 0.53±0.12 | 0.36±0.14 | 0.71±0.29 | 1.41±0.34 |
